# Prevalence of probable substance use disorders among children in Ugandan health facilities

**DOI:** 10.1186/s12889-024-17732-6

**Published:** 2024-01-29

**Authors:** Harriet Aber-Odonga, Juliet Ndimwibo Babirye, Ingunn Marie S. Engebretsen, Fred Nuwaha

**Affiliations:** 1 Makerere University College of Health Sciences School of Public Health, Kampala, P.O Box 7072, Uganda; 2 Centre for International Health, Department of Global Public Health and Primary Care, University of Bergen, Bergen, Norway

**Keywords:** Alcohol use, Substance use, Disorder, Illicit drug use, Children, Screening, Primary, Healthcare, Facilities, Uganda, Developing country, Africa

## Abstract

**Background:**

Globally, there is a concerning surge in the prevalence of substance use among adolescents and children, creating a substantial public health problem. Despite the magnitude of this issue, accessing healthcare explicitly for substance use remains challenging, even though many substance users frequently visit healthcare institutions for other health-related issues. To address this gap, proactive screening for substance use disorders has emerged as a critical strategy for identifying and engaging patients at risk of substance use. The purpose of this study was to investigate the prevalence of probable alcohol and other substance use disorders, and associated factors, among children aged 6 to 17 years old attending health facilities in Mbale, Uganda.

**Methods:**

We conducted a health facility cross-sectional study, involving 854 children aged 6–17 years. The prevalence of probable alcohol and other substance use disorders was assessed using a validated Car, Relax, Alone, Forget, Friends, Trouble (CRAFFT) screening tool. Univariable and multivariable modified Poisson regression analyses were performed using STATA 15 software.

**Results:**

The overall prevalence of probable alcohol use disorders (AUD) and other substance use disorders (SUD) was 27.8% (95% CI 1.24–1.31) while that of probable AUD alone was 25.3% (95% CI 1.22–1.28). Peer substance use (APR = 1.24, 95% CI 1.10–1.32), sibling substance use (APR = 1.14, 95% CI 1.06–1.23), catholic caregiver religion (APR = 1.07 95% CI 1.01–1.13), caregiver income of more than $128 (APR = 0.90, 95% CI 0.82–0.98), having no parental reprimand for substance use (APR = 1.05, 95% CI 1.01–1.10) and having no knowledge of how to decline an offer to use substances (APR = 1.06, 95% CI 1.01–1.12) were found to be significantly associated with probable AUD/SUD.

**Conclusions:**

Our findings suggest a high prevalence of probable AUD and SUD among children and adolescents visiting healthcare facilities for other conditions, along with a strong link between AUD and SUD prevalence and social factors. The implication for our healthcare system is to actively screen for and treat these conditions at primary healthcare facilities.

## Introduction

Alcohol and other substances use are responsible for one in every five fatalities globally each year and contribute to over 200 illness and injury conditions [1]. While alcohol consumption accounts for 6.4% of all deaths and 4.7% of all Disability Adjusted Life Years (DALYs) in the African Region [2], it is also the leading risk factor for death and disability among African male adolescents aged 15–24 years [3, 4]. Uganda has one of the highest per capita alcohol consumption rates in Sub-Saharan Africa [1] and an earlier study found that approximately 39.1% of children aged 12–24 used substances regularly [5].

Among young people, alcohol is the number one choice of substance for use and its pattern of use among this group is one of drinking too much and at too early an age [6–9]. In our specific context, evidence reveals that children commence experimentation with alcohol use as soon as they are able to hold a glass. Another study conducted within our region similarly highlighted cases of alcohol use dependence among children as young as 5 to 8 years old [10, 11]. This early initiation of substance use is associated with a greater likelihood of developing substance dependence in adulthood [12–19].

Furthermore, a systematic review found that those who begin using substances before age 13 years are much more likely to frequently drink to intoxication or die from an overdose [20, 21]. These children and adolescents also often exhibit behaviours that pose a risk to themselves and others, such as being injured in fights and suicide attempts and experimenting with risky sexual behaviour [22–25].

Despite well documented evidence that children start using alcohol and other substances early [7, 12, 13], additional evidence indicates that individuals with alcohol and substance use disorders often access the health care system for reasons other than seeking care for substance use problems [26, 27]. Many do not seek specialty treatment but they are over-represented in many general health care settings, signalling that the health care system is an important entry point for recognising and later addressing these underlying disorders [27, 28]. Some literature on the prevalence of substance use among children in the community as well as facility settings is available [11, 29–33]. Nevertheless, these studies face limitations such as small sample sizes, the exclusion of children under the age of 12—despite evidence in our context indicating earlier substance use initiation—variations in contexts, and the use of screening tools that may lack sensitivity towards children. In this study we estimated the prevalence of probable alcohol and substance use disorders among children aged 6–17 years attending care at health facilities in Mbale district using the CRAFFT screening tool.

## Methods

### Study setting and design

This health facility cross-sectional study was done in Mbale City and district between June - August 2022. Mbale is located in the mid-eastern region of Uganda and has 54 health facilities, two private hospitals and one regional referral hospital that serves the eastern region and also offers specialized mental health care. The district had an estimated population of 586,300 people in 2020 while the City had a population of about 80,723 [34]. Mbale was chosen for the study because of reports of high early initiation to alcohol and other substance use reported among children and adolescents in the district [7].

### Sample size and sampling procedure

We estimated the sample size to be 854 using the Kish Leslie (1965) formula. The prevalence of probable AUD (Alcohol Use Disorder) in our study was assumed to be 50%, as specific prevalence data for our study age category was not available in other studies. We used a 0.05 error, a design effect of 2, and accounted for a 10% non-response rate, based on previous research by Wondafrash et al. (2012).

To identify the study participants, we implemented a two-stage sampling procedure. During the first stage, all health care facilities were selected for potential inclusion. However, specialized facilities such as women’s clinics, and HIV clinics were excluded to focus on healthcare settings. This decision was made because these specialized facilities routinely screen for substance and alcohol use among their clients. Such screening practices are not as common in general health facilities, which serve a larger portion of the population. Therefore, including specialized clinics in our study could bias the findings towards better service delivery, failing to offer an accurate reflection of the conditions in general facilities. In the second stage, we employed a probability proportionate to size approach based on the patient attendance at each facility. We calculated the proportion of study participants to sample at each level using estimates of the mean daily health facility attendance for children aged 5 to 19 years, who sought care in health facilities during 2020, as obtained from the Mbale district Outpatient Department (refer to Table 1 for details).

**Table 1:** Sampling of participants per facility level

Facility level	Total no of facilities	No of selected facilities	Daily facility attendance 5–19 yrs.	% Daily attendance	Sample per level
**Mbale Regional Referral Hospital**	1	1(Govt)	32	0.07	60
**Hospital**	2	2(Private)	5*2 = 10	0.02	16
**Health Centre IV**	4	4(1 private, 3 Govt)	13*4 = 52	0.10	86
**Health Centre III**	28	All facilities	10*28 = 280	0.55	470
**Health Centre II**	22	All facilities	6*22 = 132	0.26	222
**Total**	57	57 facilities	506	1	854

At each health care facility, we utilized consecutive sampling to identify all eligible children and adolescents who visited the health facility between June and August 2022. To be eligible, the participants needed to be children or adolescents aged 6 to 17 years seeking care at the outpatient section of the health facilities. We approached both the caregivers and their children or adolescents for inclusion in the study.

#### Inclusion criteria

Consenting caregivers and assenting Children and adolescents were included in the study including emancipated minors. A caregiver was defined as the legal guardian of the child, including biological parents and adoptive relatives responsible for the child’s overall well-being.

The interviews took place after the completion of their clinical appointment and just before departing the health facility. We ensured a consecutive sampling approach, where eligible study participants were interviewed until we reached the target sample size for each facility.

### Measurements

Alcohol and other substance use was defined as; a child’s positive response (yes) to consumption of alcohol and other substances which was more than a few sips or sniffs and not just tasting. This was then categorized into three: use in their lifetime (that is, from any date in their life to date), use in the last 12 months and use in the last 30 days (current use).

The primary outcome of this study was prevalence of probable alcohol and other illicit substance use disorders among patients aged 6–17 years attending outpatient facilities. Probable Alcohol and Substance use disorder was assessed using the CRAFTT tool consisting of the six questions [35]. As a component of the broader Treat Child Alcohol Use Disorder (TREAT CAUD) project in Mbale district, Eastern Uganda, the CRAFFT tool underwent validation among 470 children aged 6–13 years. In this validation process, the tool was translated into Lumasaaba (the language commonly used in Mbale) and tailored to align with the local context. The Lumasaaba version of the CRAFFT tool exhibited strong internal consistency (Cronbach’s α = 0.86) and inter-item correlation (Spearman correlation coefficient of 0.84). It also, demonstrated optimal sensitivity (91%) and specificity (92%). The version utilized in this study was this context-adapted CRAFFT tool, and statements included in italics are components of the adapted version. We chose to use the CRAFFT screening tool since it is appropriate for screening children, and has been validated against the MINI-KID that is diagnostic. We also adopted the cut-off of more than two positive responses to the CRAFFT questions. These questions included: (1) Have you ever driven/been driven by someone using a CAR*/bicycle/motorcycle/scooter or bodaboda* while you/they were drunk, or high or had been using alcohol/drugs? (2) Do you ever use alcohol/drugs (marijuana, tobacco) to RELAX, feel better about yourself/ be able to sleep/perform better or fit in *(Not to feel shy/be accepted/ fit in group/ be same as others*? (3) Do you ever use alcohol/drugs while you are by yourself, or ALONE *(when nobody is seeing you)?* (4) Do you ever FORGET *(not remember)* things you did when you had drunk alcohol/ used other drugs? (5) Do your FAMILY *(parents, brothers, sisters, relatives, or other people who stay in your home)* or FRIENDS ever tell you that you should reduce/stop drinking/use of other drugs? (6) Have you ever gotten into TROUBLE while you were using alcohol or other drugs?

A child was considered to have probable AUD if he/she reported use of alcohol and had a positive response to 2 or more questions in the CRAFFT tool, similarly a child was considered to have probable SUD if he/she reported use of either tobacco, marijuana or any other illicit drug and had a positive response to 2 or more questions in the CRAFFT tool.

Additional data collected included: age, sex, tribe, religion, school attendance, relationship to caregiver, marital status of caregiver, education levels, household size, household income, occupation of caregiver. We also collected data on possible risk factors for substance use such as peer substance use, sibling substance use, parental substance use, child knowledge and attitude towards substance use, violence at home and child’s relationships in the home.

### Data collection, management and analysis

To collect data for this study, we used a pretested structured questionnaire. Face-to-face interviews were conducted with caregivers and their children, and hard-copy questionnaires were utilized for this purpose.

The caregiver-child exit interviews lasted between 30 and 40 min. To accommodate participants who spoke the commonly spoken local language, Lumasaba, the questionnaires were initially written in English and then translated into Lumasaba. To ensure accuracy and consistency in the questions’ meanings, a back translation of all questionnaires was performed. Before initiating data collection, we pre-tested the questionnaire in a health center of level IV facility in Kampala (located in another district). This pre-testing helped us identify and resolve any ambiguities in the questions and ensured that the questions were easily understandable by the participants.

The interviews and screening for probable AUD were conducted by 30 trained health workers. Prior to their engagement in the study, these interviewers received thorough training on the questionnaire to ensure consistency and accuracy in interpreting and administering each question. They were specifically trained on the study objectives, the CRAFFT screening and questioning techniques, and participant selection. This preparation ensured that the data collection process was conducted in a standardized and reliable manner.

We used Excel spreadsheets for double data entry, with two independent data enumerators cross-referencing each other’s work to enhance data completeness and consistency with the hard copy records. Subsequently, the data was imported into STATA software version 15 for further cleaning and statistical analysis. To provide an overview of the data, we calculated means and corresponding standard deviations for continuous characteristics, while frequencies and corresponding proportions were computed for categorical socio-demographic characteristics, risk factors for alcohol and substance use disorders, and knowledge and attitudes towards substance use.

To determine the associations between probable alcohol and other substance use disorders and independent variables, we adopted a modified Poisson regression analysis approach to estimate the prevalence ratios (PR) and their 95% confidence interval (CI). In the multivariable model, all associations with a p-value less than 0.2 from the bivariable analysis were taken into consideration. Furthermore, to evaluate the goodness of fit of our model, we conducted a Pearson chi-square goodness-of-fit test, which confirmed the model’s appropriateness and robustness in representing the data. We also tested for multicollinearity across similar variables.

### Ethical considerations

The project within which this study was conducted obtained ethical approval from the Regional Committees for Medical Research Ethics-South East Norway on 6 March 2020, with reference number 50. Additionally, the study received ethical approval from the Makerere University School of Public Health Higher Degrees Research and Ethics Committee (SPH-2022-224) and the Uganda National Council of Science and Technology (HS2182ES). To uphold participants’ rights and ensure their voluntary participation, a parent/legal guardian gave informed consent for their own participation as well as the participation of their children in this study including children who were illiterate. Additionally, after obtaining parental/legal guardian informed consent for the child to participate in the study, children were also asked to give assent to participate in the study.

## Results

### Socio demographic characteristics of respondents

In this study, we analyzed data from 834 respondents, achieving a 98% response rate by excluding 20 records with missing data. Among the children, more than half, 430 (51.7%) were aged 14 to 17, with a mean age of 12.9 (SD: 3.3), and 420 (50.9%) were male. Nearly all, 816 (98.2%) the children had attended school, with most, 610 (73.1%) currently in the first seven years of formal schooling. Caregivers aged 40 to 49 constituted 271 (33.5%), with a mean age of 39.9 (SD: 11.4). Female caregivers accounted for more than half, 561 (69.3%) of the caregiver population, while 329 (40.7%) had completed the initial seven years of formal schooling (see Table 2).

**Table 2:** Child and adolescent alcohol and substance use

Substances used	Total (834)	Male (424)	Female (410)
**Alcohol use**			
Lifetime use	329/834(39.4)	205(62.3)	124(37.7)
Last 12 months	283/834(33.9)	176(62.2)	107(37.8)
Last 30 days (current use)	197/834(23.6)	127(64.5)	70(35.5)
**Tobacco use**			
Last 12 months	40/834(4.8)	32(80)	8(20)
Last 30 days (current use)	22/834(2.6)	20(90.9)	2(9.1)
**Marijuana use**			
Last 12 months	44/834(5.3)	37(84.1)	7(15.9)
Last 30 days (current use)	33/834(4.0)	27(81.8)	6(18.2)
**Any other illicit drug use**			
Last 12 months	43/834(5.2)	37(86)	6(14)
Last 30 days (current use)	27/834(3.3)	22(81.5)	5(18.5)
Total current substance use of at least one (tobacco, marijuana, other illicit drug)	**58/834(7.0)**	**47(81)**	**11(19)**
Lifetime use of (tobacco, marijuana, other illicit drug)	127/834(15.2)	93(73.2)	34(26.8)
CRAFFT probable AUD and SUD Severity			
**Prevalence of probable AUD only**	**211/834(25.3)**	**138(65.4)**	**73(34.6)**
No AUD	623/834(74.7)	286(45.9)	337(54.1)
**Prevalence of probable SUD only**	**32/834(3.8)**	**26(81.3)**	**6(18.7)**
No SUD	802/834(96.2)	398(49.6)	404(50.4)
**Combined Prevalence (probable AUD or SUD)**	**232/834(27.8)**	**152(65.5)**	**80(34.5)**
No AUD or SUD	602/834(72.8)	272(45.2)	330(54.8)

### Prevalence of probable AUDs and SUDs

More than a third, 329 (39.4%) of children had ever consumed alcohol and more than half, 205 (62.3%) of these were male. Among those that had used other substances in the past 12 months, 40 (4.9%) had used tobacco, 40 (4.8%) had used marijuana and 44 (5.3%) had used some other illicit drug. In regard to use in the past 30 days, 197 (23.6%) of children had used alcohol, 22 (2.6%) tobacco, 33 (4.0%) marijuana and 27 (3.3%) any other illicit drug. In this screening activity using the CRAFFT tool, a quarter, 27.8% (232/834) of the study subjects met the criteria for having either probable alcohol use disorders or probable substance use disorders. Of these, 25.3% (211/834) had probable AUD and 3.8% (32/834) had probable SUD. Among the children screened with probable AUD/SUD, over two-thirds, (72.5%) were in the age range of 14–17 years. Additionally, 18.5% fell within the age group of 10–13 years, and 9% were in the 6–9 years age range. (See Table 3 for details).

**Table 3:** Sociodemographic characteristics of respondents

Variables	Frequency	Percent
Child characteristics		
**Child age (years)**		
6–9	157	18.9
10–13	245	29.5
14–17	430	51.7
**Sex**		
Male	420	50.9
Female	404	49.1
**Ever attended school**		
Yes	816	98.2
No	15	1.8
**Currently in school**		
Yes	740	89.2
No	90	10.8
**Highest level of Education**		
< 7 years	610	73.1
> 8 years	224	26.9
Caregiver characteristics		
**Age**		
14–29	141	17.5
30–39	255	31.6
40–49	271	33.5
50+	140	17.4
**Sex**		
Male	248	30.7
Female	561	69.3
**Education level**		
None	70	8.7
≤ 7 years	329	40.7
8–13 years	231	28.6
> 13 years	178	22.0
**Religious affiliation of caregiver**		
Anglican	261	32.3
Catholic	199	24.6
Pentecostal	141	17.5
SDA	47	5.8
Muslim	160	19.8
**Caregiver occupation**		
Vendor	128	15.8
Farmer	440	54.2
Salaried worker	244	30.0
**Caregiver Marital status**		
Cohabiting	101	12.6
Married	532	66.4
Divorced/separated	107	13.4
Widow	61	7.6
**Tribe**		
Lumasaaba	585	72.2
Others	225	27.8
**Family size**		
1–4	192	24.0
5–8	487	60.8
9–10+	122	15.2
**Family Type**		
Polygamous	235	29.2
Not	571	70.8
Average household income*		
0-100,000	496	59.5
100,001-250,000	139	16.7
250001-500,000	142	17.0
500,001–1,000,000+	57	6.8

*We used an exchange rate of 1USD = 3600 UGX

### Factors associated with probable child and adolescent AUD and SUD

In this study the factors we found to be associated with probable AUD/SUD included peer and sibling substance use, caregiver religion and education attainment, household income, child’s schooling level, child’s self-efficacy, knowledge and parental reprimand.

We found that children whose peers (APR = 1.24, 95% CI 1.1–1.32) and siblings (APR = 1.14, 95% CI 1.06–1.23) used alcohol and other substances had a higher chance of being users themselves (see Table 4 for details).

**Table 4:** Factors associated with probable child and adolescent alcohol and substance use disorders

Variable	Prevalence of (probable AUD or SUD) *n* = 232	Crude Prevalence ratio (95% CI)	Adjusted Prevalence ratio (95% CI)
**Child age (years)**			
6–9	21(9.0)	1	1
10–13	43(18.5)	1.0(0.9–1.1)	0.99(0.95–1.05)
14–17	168(72.5)	1.2(1.1–1.3)	1.01(0.95–1.08)
**Sex**			
Male	152(65.5)	1.1(1.0-1.2)	1.00(0.97–1.04)
**Highest level of Education**			
≤ 7 years (Ref)	141(60.7)	1	1
≥8 years	91(39.3)	1.1(1.08–1.2)	**1.05(1.01–1.11)**
Caregiver characteristics			
**Age (n-216)**			
14–29	27(12.5)	1	1
30–39	63(29.2)	1.05(0.9–1.12)	0.99(0.94–1.05)
40–49	81(37.5)	1.09(1.01–1.2)	0.98(0.93–1.04)
50+	45(20.8)	1.1(1.02–1.2)	1.00(0.94–1.06)
**Sex (n-211)**			
Male	68(31.0)	1	1
Female	151(69.0)	0.9(0.9-1.0)	0.99(0.96–1.03)
**Education level (n-219)**			
None	24(10.9)	1.1(1.02–1.24)	1.08(1.01–1.17)*
≤ 7 years	112(51.1)	1.13(1.06–1.2)	1.09(1.03–1.16)**
8–13 years	49(22.4)	1.02(0.9–1.09)	1.03(0.98–1.09)
> 13 years (Ref)	33(15.6)	1	1
**Religious affiliation of caregiver (n-219)**			
Anglican	84(36.2)	1.2(1.13–1.28)	1.02(0.97–1.07)
Catholic	80(34.5)	1.28(1.2–1.36)	1.06(1.01–1.10)*
Pentecostal	28(12.1)	1.09(1.02–1.17)	1.01(0.95–1.07)
SDA	12(5.2)	1.14(1.03–1.27)	1.02(0.92–1.13)
Muslim	15(6.5)	1	1
**Average household income (n-232)**			
0-100,000	149(64.2)	1	1
100,001-250,000	38(16.4)	0.98(0.91–1.05)	0.99(0.94–1.05)
250001-500,000	35(15.1)	0.95(0.89–1.02)	0.98(0.92–1.04)
500,001–1,000,000+	10(4.3)	0.9(0.82–0.99)	0.90(0.82–0.98)*
**Health Facility (n-232)**			
RRH	12(5.2)	1	1
HCIV	27(11.6)	1.14(1.02–1.28)	1.12(1.01–1.24)*
HC III	159(68.5)	1.06(0.97–1.16)	1.14(1.03–1.24)**
HC II	34(14.6)	1.07(0.96–1.18)	1.07(0.96–1.18)
Peer alcohol and substance use (Yes) have to include no?	210(90.5)	1.51(1.46–1.57)	1.24(1.1–1.32)***
Sibling alcohol and substance use (Yes)	191(82.3)	1.51(1.44–1.57)	1.14(1.06–1.23)***
Caregiver SU	151(65.1)	1.29(1.23–1.35	1.01(0.96–1.07)
Caregiver alcohol use (Yes)	126(54.3)	1.28(1.22–1.34)	0.96(0.91–1.02)
Caregiver tobacco use (Yes)	30(12.9)	1.28(1.17–1.40)	0.96(0.88–1.05)
Caregiver illicit drug use (Yes)	28(12.0)	1.33(1.22–1.45)	1.08(0.98–1.19)
Relationship with your mother/female caregiver			
Good	167(71.9)	1	1
Bad	64(27.6)	1.35(1.27–1.44)	1.02(0.95–1.10)
Relationship with your father/male caregiver			
Good	153(65.9)	1	1
Bad	77(33.1)	1.29(1.22–1.37)	1.02(0.96–1.08)
Do adults or parents fight a lot at your home?			
No	155(67.3)	1	1
Yes	76(32.7)	1.08(1.02–1.14)	0.98(0.93–1.02)
If one of your friends offered you a drink of alcohol or drugs, would you take it?			
No	79(34.3)	1	1
Yes	151(65.7)	1.41(1.35–1.48)	1.07(1.01–1.14)*
Ever learned about effects of substance use; use on decision making			
Yes	85(36.6)	0.94(0.89–0.98)	0.94(0.87–1.02)
No	147(63.3)	1	1
Ever learned about how to tell someone you did not want to take alcohol or other drugs			
Yes	121(52.2)	1	1
No	111(47.8)	0.97(0.93–1.02)	1.06(1.01–1.12)*
Parental punishment for alcohol/substance use			
Yes	125(54.1)	1	1
No	106(45.9)	1.20(1.14–1.26)	1.05(1.01–1.10)*
Ever talked with your friends about alcohol or drugs(yes)	140(60.3)	1.11(1.06–1.17)	1.05(1.01–1.09)*

We also found that the factors associated with overall probable AUD or SUD included children who had attained at least 8 years of school or more (APR = 1.05, 95% CI 1.004–1.11), catholic caregiver religion (APR = 1.06, 95% CI 1.01–1.10), children whose caregivers either had no schooling (APR = 1.08, 95% CI 1.001–1.17) or had only completed 7 years of formal schooling (APR = 1.09, 95% CI 1.03–1.16) and children who attended middle level health facilities (HC III) (APR = 1.14, 95% CI 1.03–1.24). Furthermore, we found that children whose parents earned more than $128 (APR = 0.90, 95% CI 0.82–0.98) were less likely to have probable substance use disorders compared to those who earned less than $28. Children who agreed that they would consume alcohol/other substances if a friend recommended it (APR = 1.07 95% CI 1.004–1.14), those who had never learned to say no to substance use (APR = 1.06, 95% CI 1.01–1.12),those who were not afraid of parental punishment for substance use (APR = 1.05, 95% CI 1.00-1.10) and those who spoke to a friend about substance use (APR = 1.05, 95% CI 1.002–1.09) had a higher prevalence of probable AUD or SUD (See Table 4 for details).

## Discussion

To the best of our knowledge, this is the first study examining substance use disorder prevalence among children aged 6–17 years old receiving care at facilities using the CRAFFT screening tool. The overall prevalence of probable AUD or SUD in our study was 27.8%, which is comparable to a study done among 12-18year-olds in Chicago that found a 27.9% prevalence of substance use disorder in clinical settings [36]. In contrast, Fischer and Grange (2007) discovered a prevalence of 11% in an outpatient sample of psychiatrically referred teenagers aged 13–19 years in Chicago, while Mann et al. (2014) reported a prevalence of 6% and Kaggwa et al. found a prevalence of 7.2%; these figures were lower than what we found in our study. Possible reasons for these discrepancies include variations in our contexts, the age groups studied, and differences in the tools employed. Both Mann and Fischer used the DSM IV/V while Kaggwa used the ICD-11 which are all diagnostic tests, unlike the CRAFFT utilized in our study, which is a screening tool. This divergence in diagnostic approaches may account for the observed smaller proportion in their studies compared to our findings [37, 38].

Factors associated with probable Child and adolescent AUD and SUD prevalence.

### Peer and sibling substance use

In our study, peer substance use emerged as a significant predictor of probable Alcohol Use Disorder (AUD) and Substance Use Disorder (SUD), consistent with findings from prior research. Studies have consistently shown that peer substance use strongly predicts substance use during early adolescence [39, 40]. Children, in particular, are highly susceptible to peer influence regarding substance use [41], especially as they strive to fit in with their peers during this developmental stage.

Social network analyses indicate that adolescents are especially vulnerable to peer contagion effects, but certain protective factors can counteract these influences. Adult monitoring, supervision, positive parenting, structure, and self-regulation have been identified as crucial protective elements [42, 43].

Our findings also revealed a positive connection between children who discussed substance use with their peers and those who expressed willingness to drink alcohol if offered by a friend. This underscores the powerful impact that peers have on one another in shaping attitudes and behaviors related to substance use [5, 44–47].

Sibling substance use emerged as the second strongest predictor of both probable Alcohol Use Disorder (AUD) and probable Substance Use Disorder (SUD) prevalence in our study, corroborating findings from previous research. Studies have consistently shown that older sibling substance use has a direct effect on younger sibling usage [48]. Similarly, Whiteman et al. (2013) demonstrated a positive association between older siblings’ alcohol and substance use and the patterns of use in younger siblings.

The link between sibling substance use can be attributed to the influence they exert on each other through their interpersonal dynamics and the opportunities they provide for substance use involvement [49–51]. Additionally, the impact of older siblings’ alcohol use may be particularly potent when combined with their admiration and overlapping peer networks. Recent research has also highlighted that older siblings serve as critical and unique socialization agents, influencing younger siblings’ expectations and intentions regarding substance use [43, 46, 52, 53].

### Caregiver education level

The prevalence of probable child AUD was higher among children whose caregivers had either no education or only attained lower level of education. This could be because literature suggests that children of educated parents are most likely better aware of the dangers of alcohol consumption, which may discourage binge drinking and/or encourage more moderate but frequent use [54].

### Religion

Children with Catholic caregivers had a higher prevalence of probable AUD than children with Muslim caregivers in our study; this can be explained by the fact that children who belong to homes that adhere to religions that prohibit the intake of alcohol and other substances will most likely also prohibit the children. It is crucial to note that this doesn’t imply a direct correlation; rather, evidence suggests that low levels of religiosity are associated with adolescent substance use and abuse [55–57]. This indicates that children with strong religious affiliations, be it Catholic or Muslim, are more inclined to either abstain or engage in alcohol use based on their personal commitment to their faith. As demonstrated by Oetting et al., religion plays a role in building resilience in adolescence by reinforcing “primary socialization sources” such as family, friends, and school. In an environment that is more accepting of alcohol use, a child may find it easier to experiment with alcohol, contrasting a hostile environment that threatens severe punishment or potential excommunication [58].

### Facility level

Our study revealed that children seeking care from mid-level facilities had a higher likelihood of experiencing probable Alcohol Use Disorder (AUD) and Substance Use Disorder (SUD) compared to those seeking care at the regional referral hospital. This finding suggests that smaller facilities may be more accessible to these children, making them potential entry points for targeted interventions to address the problem.

### Parental reprimand

In addition, our findings indicated that fear of parental punishment or reprimand was associated with an increased prevalence of probable SUD. this is consistent with a study done among Latino Youth in the US that found that clear rules set by parents regarding substance use and the belief that certain consequences are attached to behaviors were associated with a lower prevalence of probable SUD [62].

### Child’s education level

Furthermore, we observed that children attending secondary school were more likely to have probable Substance Use Disorder (SUD). This finding is consistent with a study conducted among adolescents in Indonesia, which reported a significant association between Alcohol Use Disorder (AUD) prevalence and higher education levels [63, 64]. The higher prevalence of probable SUD in secondary school children may also be attributed to their older age, as studies have demonstrated that older age is linked with an increased risk of substance use problems [36]. Similarly, a study conducted in Uganda revealed a higher prevalence of SUD among older adolescents [38]. This further supports the notion that older age groups are more vulnerable to substance use issues.

### Household income

Our study findings indicated that high household income was associated with a lower prevalence of probable Substance Use Disorder (SUD). This result aligns with previous literature that has consistently shown a link between lower socioeconomic status and higher rates of substance use problems [65]. However, it is essential to acknowledge that Humnesky proposed a contrasting perspective. According to Humnesky (2010), illicit substances’ demand and their sensitivity to price could lead to an increase in substance use as income levels rise [66]. While these contrasting viewpoints exist, our study’s alignment with previous research suggests that higher household income may serve as a protective factor against SUD. Nonetheless, the complex relationship between income, substance use, and other underlying factors necessitates further exploration and understanding.

### Child’s self-efficacy

Children who demonstrated awareness about ways of declining substance use exhibited a lower prevalence of probable Substance Use Disorder (SUD) in our study. This finding highlights the significance of self-efficacy in influencing substance use behaviours, which is consistent with research conducted in Uganda. Studies in Uganda revealed that adolescents with high levels of self-confidence were less likely to engage in substance use [5, 46, 67].

The association between self-efficacy and reduced probable SUD prevalence underscores the importance of empowering children with the knowledge and skills to resist substance use temptations. Enhancing self-confidence and promoting a sense of control can serve as protective factors against the initiation and continuation of substance use.

The findings of this study hold significant implications for preventive programs aimed at reducing alcohol and substance use among children and adolescents. One key implication is that the influence of peer and sibling interactions, as well as larger networks, can impact substance use long before traditional prevention efforts typically begin. While many prevention programs focus on middle adolescent years, this study suggests that addressing key associated factors early is crucial.

To be effective, prevention campaigns should target factors such as resisting peer influences. Initiating such campaigns at an early age, even as young as 6 years old, is essential. Additionally, family sensitization should be conducted not only in schools but also within the community and health facilities. Understanding the prevalence of alcohol and substance use disorders among children and adolescents, along with the strong association between peer and sibling networks, individual factors like low awareness and self-efficacy, and family-related factors (e.g., education, income, household relationships, and family monitoring), underscores the importance of substance use programs that encompass support for children through these networks.

### Study limitations

Despite the valuable insights gained from this facility-based study, it is important to acknowledge its limitations, which may impact the generalizability of the results to the broader population. One notable limitation is our inability to estimate the true magnitude of substance use disorder prevalence in the general population due to the study’s setting. As a result, caution should be exercised when applying these findings to other populations. Furthermore, it is possible that participants may have underreported their substance use despite the use of a structured interview. This self-reporting bias could potentially impact the accuracy of prevalence estimates. Additional underreporting might have occurred due to the interviews being conducted by a health worker in a facility close to the participants’ households. This proximity could have potentially introduced social desirability bias into the responses.

Additionally, the use of the CRAFFT tool, while child-appropriate and useful for assessing probable substance use disorder, limited our ability to differentiate between individual substances. Nevertheless, we did assess CRAFFT separately for alcohol use and SUD to gain more specific insights.

The cross-sectional design of the study also poses limitations as it prevents us from examining the timing of substance use development. A longitudinal approach would have provided more comprehensive insights into the predictors of AUD and SUD prevalence in this population. Notwithstanding these limitations, this study is novel as it is the first to examine patterns of probable alcohol and substance use disorder prevalence in a clinical setting across a diverse age group [6–17] using the child-appropriate CRAFFT tool. Additionally, by examining this prevalence across all facility levels, we captured a more comprehensive picture within our health system.

Future research should focus on longitudinal studies to assess the development of probable child AUD and SUD over time. Moreover, exploring preventive interventions that incorporate the role of social influences in both treatment and practice parameters could be instrumental in effectively addressing substance use disorders among children and adolescents. By overcoming these limitations and building on the strengths of this study, we can advance our understanding and improve the outcomes for this vulnerable population.

## Conclusions

This study deviates from the common approach of conducting prevalence studies in community settings, as we specifically focused on health facilities. Our primary aim was to provide evidence on the importance of screening for substance use among children seeking care in these facilities. Our findings revealed a high prevalence of probable Alcohol Use Disorder (AUD) and Substance Use Disorder (SUD) among children attending health facilities. Moreover, we identified several key predictors of these disorders, including peer and sibling substance use, lower education attainment of the caregiver, Catholic caregiver religion, caregiver income exceeding 28 USD, a poor relationship between the child and their male caregiver, as well as children lacking awareness of substance effects and how to decline offers to use substances.

These results highlight the hidden problem of alcohol and substance use that prevails in our health system. It emphasizes the urgent need for a comprehensive and early approach to prevention to effectively address substance use issues among young populations. Targeting peer influences and supporting children through various networks can pave the way for impactful preventive programs, addressing the root causes of alcohol and substance use and promoting healthier behaviors among children and adolescents. We also advocate for policy considerations that involve the integration of rapid screening tools like the CRAFFT into clinical care. Furthermore, we propose the implementation of mandatory routine screening for alcohol and substance use among children and adolescents as part of their standard health care.

## Data Availability

The datasets generated and/or analysed during the current study are available upon request. The first author, HAO can be contacted for access to the datasets using this email address haber@musph.ac.ug.

## Abbreviations

AUD: Alcohol Use disorders
LMICs: Low- and Middle-Income Countries
SU: Substance Use
SUD: Substance Use disorders
WHO: World Health Organization
